# Smart Waste Collection System with Low Consumption LoRaWAN Nodes and Route Optimization

**DOI:** 10.3390/s18051465

**Published:** 2018-05-08

**Authors:** Álvaro Lozano, Javier Caridad, Juan Francisco De Paz, Gabriel Villarrubia González, Javier Bajo

**Affiliations:** 1 Faculty of Science, University of Salamanca, Plaza de la Merced s/n, 37002 Salamanca, Spain; jch@usal.es (J.C.); fcofds@usal.es (J.F.D.P.); gvg@usal.es (G.V.G.); 2 Department of Artificial Intelligence, Polytechnic University of Madrid, Campus Montegancedo s/n, Boadilla del Monte, 28660 Madrid, Spain; jbajo@fi.upm.es

**Keywords:** smart waste collection, low powered sensors, wireless sensor networks, data analytics, decision-making, LoRaWAN, open data, VRP, CVRP

## Abstract

New solutions for managing waste have emerged due to the rise of Smart Cities and the Internet of Things. These solutions can also be applied in rural environments, but they require the deployment of a low cost and low consumption sensor network which can be used by different applications. Wireless technologies such as LoRa and low consumption microcontrollers, such as the SAM L21 family make the implementation and deployment of this kind of sensor network possible. This paper introduces a waste monitoring and management platform used in rural environments. A prototype of a low consumption wireless node is developed to obtain measurements of the weight, filling volume and temperature of a waste container. This monitoring allows the progressive filling data of every town container to be gathered and analysed as well as creating alerts in case of incidence. The platform features a module for optimising waste collection routes. This module dynamically generates routes from data obtained through the deployed nodes to save energy, time and consequently, costs. It also features a mobile application for the collection fleet which guides every driver through the best route—previously calculated for each journey. This paper presents a case study performed in the region of Salamanca to evaluate the efficiency and the viability of the system’s implementation. Data used for this case study come from open data sources, the report of the Castilla y León waste management plan and data from public tender procedures in the region of Salamanca. The results of the case study show a developed node with a great lifetime of operation, a large coverage with small deployment of antennas in the region, and a route optimization system which uses weight and volume measured by the node, and provides savings in cost, time and workforce compared to a static collection route approach.

## 1. Introduction

Nowadays, more and more cities are implementing new systems based on the Internet of Things (IoT) to obtain new data about the city, offer new services and optimize the energetic efficiency. These cities use a Smart Cities model [1] which aims to achieve more sustainable cities and make cities better places to live in. Applications developed for Smart Cities include applications for citizen security and control of people flow in cities [2], vehicle parking [3], getting information about accessible places [4], managing energy of houses [5] and public lighting [6] together with the use of smart grids, water management, waste management, health services, logistics, and a long list of other domains.

These applications can be used in medium and big cities as well as in rural and suburban areas, where this and other kinds of applications based on the IoT are more commonly found. Other applications related to smart farming [7] and the cattle industry [8] have been developed in this domain.

New smart sensors have emerged to develop this kind of projects, allowing the use of the concept “IoT” and the connection between daily used objects and the digital world. These sensors or nodes are commonly grouped into the well-known Wireless Sensors Networks (WSN) [9]. In the IoT and Ambient Intelligence (AmI) contexts [10], these objects currently equipped with sensors and actuators must naturally interact with the user. In addition, the use of wireless communication technologies makes the deployment of these elements easier in both internal and external spaces. WSN deployed in cities can equally be employed in suburban and rural places, but the economic capacities of these places are not enough to allow their implementation. This is the reason why it is essential to equip rural areas with WSN based on low-cost, little maintenance and energy-efficient technologies—for sensors as well as infrastructure—to perform the investment.

Among the previously-proposed diverse applications for Smart Cities, the use of an intelligent system of waste management is especially useful in rural areas. Intelligent management comprises monitoring of the filling volume, weight and temperature of waste containers, management of the fleet of collecting vehicles, estimations of the best routes to collect waste and management of alerts in case of an incident in a container. 

Waste collection in towns is usually delegated to municipality groupings or is directly managed by the regional administration. The routes currently performed in the large majority of the regions are static so that the collection trucks cover predefined routes whether the containers are filled or not. Efficiently managing routes and trucks required for collecting all waste in a region allows savings in fuel, workforce and maintenance of the vehicle fleet. Journeys from one town in a region to another may sometimes be several kilometers long and skipping some towns may mean important savings on fuel and time over a year.

This work presents an intelligent platform to manage waste. It consists of a network of low-cost, low energy consumption and wireless intelligent sensors, a platform with a fleet management system, a system to optimize collection routes, a monitoring web application and a mobile application to guide and track the employees of the enterprise in charge of collecting waste.

This paper is structured as follows: Section 2 presents the state of the art of sensors currently employed in the literature for smart waste collection, analyses the sensor networks that can be used to design these platforms, and describes Vehicle Routing Problems (VRP) related to collection route optimization. Section 3 presents the general architecture of the system and explains its most important components: the developed node, the analysis of the employed sensor network as well as the system and the process of route optimization. Section 4 exposes a case study performed in the region of Salamanca, Spain. This case study evaluates the developed prototype and the deployment of a WSN in that region. It also describes the feasibility of the platform by using open data from municipalities and compares the use of permanent routes and dynamic routes calculated by optimization algorithms to evaluate savings. Finally, Section 5 exposes a discussion about results in node and sensor consumption tests, the selected network coverage and the proposed system of route optimization.

## 2. State of the Art 

The use of WSN in urban and suburban areas includes very different applications as Rashid et al. described in their literature survey work [11]. Among those applications, there are several works related to the efficient management of waste in cities [12,13,14]. Longhi et al. [12] proposed a system based on WSN employing the IEEE 802.15.4 technology in their nodes. This system also measures the filling level of the containers with an ultrasonic sensor, but they only focused on the collection of the data and they did not describe the route optimization problem in depth. Gutierrez et al. [13] presented a similar work. However, they employed WiFi as the access network interface. Catania et al. [15] presented another approach where they included weight sensors, although they only used them for an interaction with the user, not for route optimization. Medvedev et al. [14] presented an optimization approach for waste collection, but they did not measure the weight of the containers or use it in their optimization system. 

Apart from these works, several solutions are available in the market, such as the ones provided by companies like Enevo [16], SmartBin [17] and SenseDumpster [18] among others. These companies offer devices that are able to measure the filling level of containers, mainly with ultrasonic sensors. However, as they do not measure weight with their devices, they do not consider it for the route optimization systems. There are other systems, like BigBelly [19], which provides a container with built-in sensors as a product.

The following sections present (1) several approaches used in the literature for obtaining different types of data, such as volume and weight, (2) the WSN that can be employed for these kinds of smart waste systems and (3) a review of the Capacitated Vehicle Routing Problem (CVRP) used in this work in order to optimize the collection routes.

### 2.1. Volume and Weight Sensors

The main sensors currently applied in smart waste systems can provide information about the waste volume of a container. These transducers may be capacitive [20], ultrasonic [21], infrared, Time of Flight (ToF) [22], or Guided wave radar (GWR). Each one of these transducers has different features that must be considered, such as range, measuring accuracy and amplitude of the working angle (described in Table 1).

The volume level estimations of the waste in a container are based on a measure of distance between the upper side of the container and the surface of the waste. Given the extent of the measurement calculated by sensors (Table 1), one of the best options is the ultrasonic sensor because it provides data from a wider area than that provided by others.

However, when carrying waste, the truck grinds and crushes the content so the waste volume of the container is highly reduced. Once waste is in the truck, the problem lies in the total weight of it, as road safety regulations do not allow trucks with excess weight to travel.

Regarding the weight of measuring containers, load cells have been traditionally used for this purpose [23,24]. The operation of load cells is based on strain gauges which are sensors using the piezoresistive effect (the electrical resistance variation produced by an effort or stress on a material) of their built-in materials. Load cells provide a data accuracy of 0.03% to 0.25%. They are suitable for virtually every industry application.

Sensors intended to measure volume and weight in this context must additionally provide great efficiency and autonomy. Complex nodes meet this requirement through the different sensors and components they include, such as the communication network employed by the device which is essential for the final consumption results of the device. 

### 2.2. Wireless Sensors Networks

Wireless sensors networks have become a crucial element in the process of sensor development for smart waste systems [25,26]. Apart from the different transducers used to measure data, one of the main purpose of sensors is to establish a level of communication that is effective over as great of a distance as possible as well as being energy-efficient to allow the battery lives of the final nodes to be increased. These two features are greatly determined by the communication networks employed in device developments. Previous literature has presented works using traditional communication networks, such as General Packet Radio Service (GPRS) cellular networks [27,28]. These networks are distinguished by their large deployment, which virtually guarantees their availability but presents a clear problem with battery consumption [29]. Also remarkable is the way that current Wireless Personal Area Networks (WPAN) try to adapt themselves to the requirements previously indicated, such as Bluetooth 5, which duplicates the range and reduces the consumption [30]. Previous literature has also included adapted specifications, such as the IEEE 802.11 ah specification, also called Wi-Fi HaLow [31]. These specifications theoretically reach connection ranges of up to 1 km when operating at lower frequencies, like 900 MHz. This technology is available but is not being used nowadays; this could be due to the fact that there are several competing technologies available in the market that better address the needs of large connection ranges [32].

ZigBee [33] is a very mature technology, with a range from 10 up to 100 m, but this technology can reach large distances by passing messages through a mesh network. Version ZigBee IP [34] allows the routing of different nodes through IPv6. However, ZigBee is a technology that is strongly focused on home automation instead of reaching great coverage distances.

Meanwhile, new communication technologies are emerging, and they are specifically designed to establish great distance communications with low bit rates and low energy consumption. These groups of networks are known as LPWAN (Low Powered Wide Area Networks) [35]. All of them are designed to reach great distances and have really low consumption so that nodes using these networks have battery lives of years. The most important technologies in this domain are Sigfox, LoRaWAN, and Narrowband Internet of Thing (NB-IoT) [36]. 

Sigfox [37] is an Ultra Narrow Band (UNB) communication network [38]. It has a marketing model similar to the one of a traditional enterprise of communication where an operator provides a signal to a big area or country and gets paid for access and transfers. The operator could be the Sigfox company itself (such as in France, Germany and Spain [39]) or other companies belonging to the Sigfox Partner Network [39] (such as IoTNet [40] in Croatia or VT-IoT [41] in Ireland).

Regarding LoRa [42,43], on one hand, it is a proprietary technology based on chirp spread spectrum (CSS) radio modulation. Semtech acquired this technology in 2012, and it currently produces transceivers which use LoRa. On the other hand, LoRaWAN [44] is a media access control layer protocol, employed for managing communication between LPWAN gateways and node devices. It defines system architecture and it is maintained by the LoRa Alliance [45]. LoRaWAN uses the physical layer protocol defined by LoRa.

The LoRaWAN specification is open and in contrast to Sigfox, a LoRaWAN network can be deployed free of charge for license or data transfer. Multiple platforms based on the LoRaWAN specification are available in the market, like Actility [46], Loriot [47], LoraServer.io [47] and Senet [48], among others.

NB-IoT [49] is an LPWAN new technology for the Internet of Things developed by the 3rd Generation Partnership Project (3GPP) [50]. NB-IoT technology employs a licensed network spectrum currently used by different Internet Service Providers (ISPs). The network can be deployed in two different ways: “in-band” or “standalone” deployment. The “in-band” deployment takes place in the spectrum allocated to Long Term Evolution (LTE), using resource blocks within the LTE carrier (even their guard-bands). The other deployment, the “standalone”, implies the use of a dedicated spectrum. NB-IoT uses the Orthogonal Frequency-Division Multiple Access (OFDMA) modulation for the downstream channel and Single Carrier Frequency Division Multiple Access (SC-FDMA) for the upstream channel. Its main advantage, in terms of communication, over other LWPANs is that it is well suited for applications that need to have minimal latency and are required to communicate more frequently [35]. It is currently being deployed throughout Europe [51], but its availability is being reduced to urban areas where various functional tests are being carried out. These provide the network, and the connection is set through classic subscription services.

Besides the technologies previously indicated, there are more technologies that are starting to become available in Europe [52], such as Random Phase Multiple Access (RPMA) by Ingenu [53,54] and Weightless of Weightless SIG [55].

### 2.3. CVRP

Regarding the selection of the waste collection route in a smart waste system, the search for an ideal waste collection route(s) is considered an optimization problem which requires the reduction of collection costs. These costs are related to the covered distance of each truck and the required number of trucks. This kind of problem is known in the literature as a Vehicle Routing Problem (VRP) [56,57]. This name is used for a whole type of problems aimed at finding a number of routes for a group of vehicles placed in a depot. They must meet the demands of a specific number of customers who are geographically spread. The purpose of a VRP is to specify the distribution/collection routes for these customers (each one has a specific demand) while reducing the cost of the vehicles’ routes, which start and end at the same depot. The original VRP is also known as the Capacited Vehicle Routing Problem and includes constraints, for example, every vehicle in one fleet has a permanent and uniform load and there is one depot. 

The original problem has several variants [57,58] which add constraints regarding real life problems, such as collections and deliveries (VRP with Pickups and Delivery [59]), time windows (VRP with Time Windows [60]), heterogeneous fleets (Heterogeneous Fleet VRP or Mixed Fleet VRP [61]) and more. 

**Figure 1:**
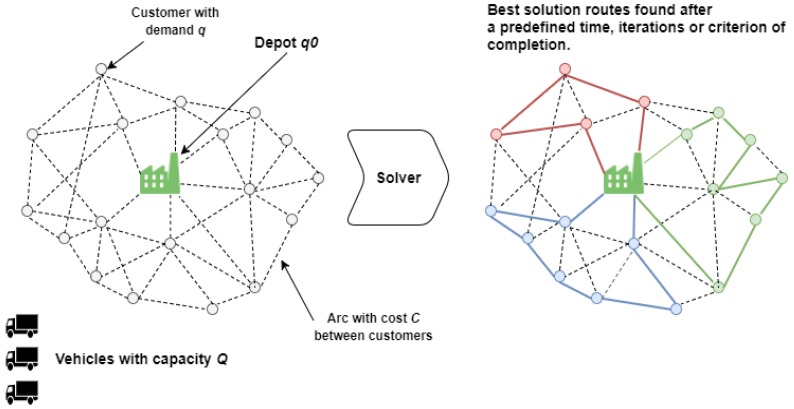
This figure shows a graphic representation of the input of a Capacitated Vehicle Routing Problem (CVRP) problem and the output route solutions.

Figure 1 shows the approach of a classic CVRP with a homogeneous fleet. This is formally defined with the following description: given G(V,A), an undirected graph where V indicates a set of n+1 vertices. A is a set of arcs, defined as $A=\left\{\left(v_{i},v_{j}\right):\;v_{i},v_{j}\;\in V,\;i\neq j\;\right\}$. V is a set (V={0,1,2,…,n}) that defines the number of customers (in this case, containers). Each $\left(v_{i},v_{j}\right)$ of set A has an associated cost (not negative) ($C_{ij}$). Each customer of the set {1,…,n} has a demand (in this case, the quantity of waste) ($q_{i}$) that must be collected and transported to the depot ($q_{0}$). The demand of the depot is always 0, so $q_{0}=0$. A set (m) of vehicles with the same capacity (Q) (if the fleet has different capacities, the problem would be another kind of VRP problem) must be employed to collect waste of n customers (containers). The m vehicles must start and end their routes in the depot. A route is defined as a lower cost cycle of the graph (G) that passes through the depot and the total demand of the set of vertices must not exceed the total capacity of the vehicle. The purpose of the problem is to reduce the distance, time or cost of the m vehicles while meeting the following requirements: (1) the depot is the start and end point of every route; (2) every customer is visited only once by just one vehicle; and (3) the total demand of each route does not exceed the capacity (Q). 

The classic CVRP is stated as follows:

(1) $$\min\sum^{\text{​}}c_{ij}x_{ij},$$

with $\left(i,j\right)\in V,\;V=\left\{0,1,2,3\ldots ,n\right\},\;x_{ij}\in \left\{0,1\right\},$ subject to

(2) $$\sum^{\text{​}}x_{0j}=m,$$

(3) $$\sum^{\text{​}}x_{i0}=m,$$

(4) $$\underset{j=1}{\sum }x_{ij}=1\;\left(i=1,\ldots ,n\right),$$

(5) $$\sum_{j=1}x_{ij}=1\;\left(j=1,\ldots ,n\right),$$

(6) m≥1.

The target function, Equation (1), is the total cost of the solution. The constraints of Equations (2) and (3) indicate that m is the number of vehicles employed for the solutions and state that all those vehicles must return to the depot. The constraints of Equations (4) and (5) turn each client into intermediate nodes of a route, and the constraint of Equation (6) states that the vehicle fleet employed must be greater than 1.

Regarding the solution, VRP and its derivatives are NP-hard problems, which means that the bigger the problem is, the greater the computational complexity to resolve it becomes. There are several solution-searching methods for these problems in the literature [62], such as (1) exact approaches which explore exhaustively explore the search space until the best solution is found. These can be branch and bound [63] or branch cut and price [64]. (2) Heuristic methods perform a limited exploration of the search space and usually deliver good quality results in small computing times [65] and (3) meta Heuristic methods are generic methods of exploration in the solution space for searching and optimization problems. These methods provide a design line adaptable to each context, and they can generate more efficient algorithms. These algorithms—Ant algorithms [66], Constraint Programming [67,68], Genetic Algorithms [69] and Tabu Search [70,71], among others—usually work better than heuristic methods.

## 3. System Proposed

### 3.1. System Architecture

**Figure 2:**
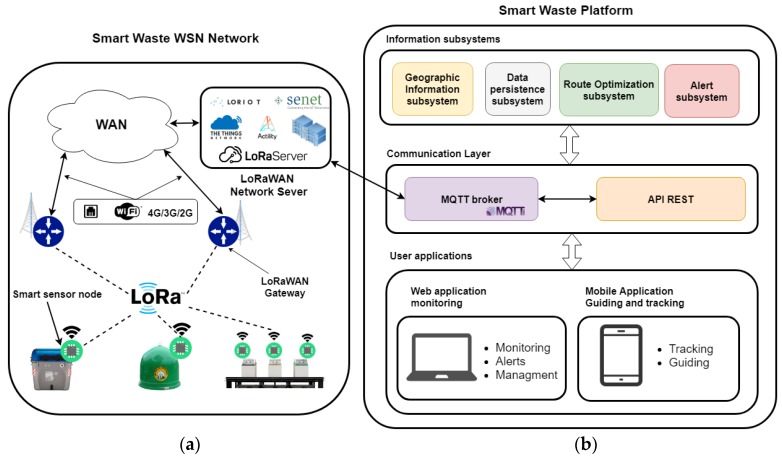
Main diagram of the system’s architecture. Wireless Sensors Network (WSN) of smart developed sensors (**a**) and the diagram of the information systems, communication and user applications (**b**).

Below is a general description of the architecture of the proposed system. Its parts and functions are explained later. Figure 2 shows a general diagram of the proposed system. On the left side (a), there is a representation of the WSN composed by the developed nodes, and their connection with the platform is shown on the right side (b). This side also presents an image of the different subsystems, communication protocols and applications provided by the system.

The platform includes four subsystems of information:

- Geographic Information Subsystem: this subsystem of the architecture stores geographic data regarding the region where the nodes are deployed. These geographic data are mainly related to the information about the routes between node locations in the system and their geocoding information. The services of this system are provided through a REST API (Application Programming Interface) to other subsystems.
- Data persistence subsystem: This system stores the information related to the management of the collection platform (data from the sensors and the deployed network, vehicles and the routes they perform, users, etc.).
- Alert subsystem: This system manages the incidences related to the information obtained through the container sensors and the vehicle fleet. They notify possible events that may happen in both the container and the vehicle fleet.
- Route optimization system: This system is responsible for searching the best collection routes for the vehicle fleet using the information obtained from the sensors.

Subsystems inside the platform use Message Queuing Telemetry Transport (MQTT) and REST web services as communication protocols. The platform also includes two user applications which employ the communication protocols and the information subsystems previously mentioned. Described below are all the components of the system emphasizing the developed node as well as the searching process to calculate the best routes for the vehicle fleet using the sensors’ data.

### 3.2. Developed Device

We decided to develop our own device instead of using other boards currently in the market, such as RN2483 LoRa Mote [72] or Waspmote (belonging to Libelium [73]) as the device base, because choosing each component separately—from microcontroller to each one of the sensors—allows the efficiency of the device to be increased, resulting in a greater battery life. Furthermore, this approach enables a solution to be provided which meets the specific requirements regarding the system’s data acquisition using the most suitable sensors for each kind of data.

**Figure 3:**
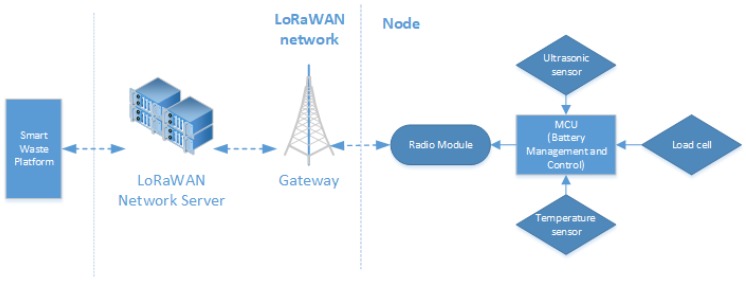
Modules of the developed node on the right side and its communication with the platform on the left side

The device is designed to obtain data from three environment variables: estimated filling volume of containers, weight of the waste inside them and their room temperatures. In contrast to other works [25] where only the estimated filling volume has been calculated, this study aimed to develop a device able to obtain useful data for optimizing routes, such as weight, and for future studies and applications, such as temperature. Figure 3 (right side) presents the device, which has several previously indicated function modules to obtain data (ultrasonic sensor, load cell, temperature sensor) as well as to manage battery consumption and control the device to obtain these data (microcontroller unit, MCU). Figure 3 (left side) describes the communication between the device and the network employing radio module.

**Figure 4:**
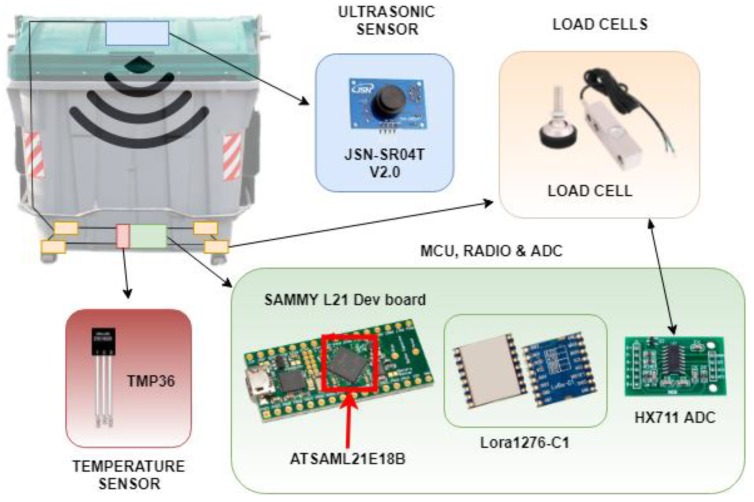
Node modules’ locations and dev board for prototyping.

The proposed node uses the container frames as the supporting platform. Figure 4 shows the location of every module (previously mentioned) inside the container, and sensors and microcontrollers are employed on each module. The device frame with the MCU, the temperature sensor, and the radio module are located in the lower section, and load cells measuring waste weight are located in the container legs. The only element located in the upper section of the container is the ultrasonic sensor measuring the estimated volume of waste.

The sensors employed for developing the device and how they operate are explained below.

JSN-SR04T V2.0 is the selected ultrasonic sensor [74]. It is a waterproof ultrasonic transceiver with an activity range between 20 and 4 m, an accuracy of 3 mm and a measuring angle of 20°. This measure is calculated by stimulating a pinger and measuring how long the stimulation takes to return to the receiver. When the signal is received, distance is calculated, as indicated in Equation (7):

(7) $$d=\frac{T*v_{s}}{2},$$

where d is the rough distance from the device to the first surface reached by the sent ultrasonic wave, T indicates the time elapsed since the release of the sound until the reception of its echo, and $v_{s}$ is the speed of the sound.

**Figure 5:**
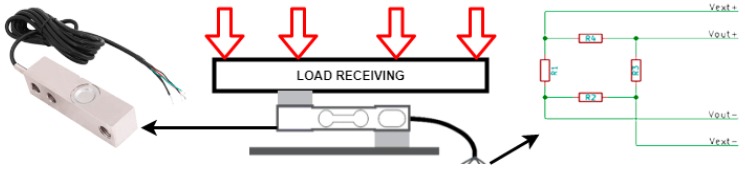
Load cell diagram and electric circuit

Container weight is calculated by using four load cells attached to the lower section and legs of the container. This kind of sensor is based on Wheatstone bridge operating principles. In Figure 5, we can see how a load cell works and its electrical circuit.

When changing weight on the transducer, the resistance is modified due to its intrinsic features. Given that it is subject to a continuous voltage difference $\left(V_{ext+}-{\;V}_{\mathrm{ext}-}\right)$, the generated voltage difference can be calculated with Equation (8):

(8) $$V_{\mathrm{out}}=V_{\mathrm{ext}}*\;\left(\frac{R_{3}}{R_{3}+R_{4}}-\frac{R_{2}}{R_{1}+R_{2}}\right).$$

As these changes in measurements are really small, an analog-digital amplifier (24-bit resolution HX711 model) is employed to obtain enough accuracy and range (it is shown in Figure 4 along with the MCU and the radio module). This amplifier receives load transducers’ signals balanced in parallel and converts the small variations into digital measurements that are sent to the microcontroller. Due to these voltage changes, weight can be calculated by using the container weight when it is empty and calibrating the voltage difference with a known weight value.

Regarding the temperature, it is measured by TMP36, an analog sensor. This sensor provides results with an accuracy of 1 °C. The temperature accuracy is not crucial for the developed sensor, but the protocol for communicating (analog in this case) is a key element.

The essential element of the developed node is the microcontroller, the Atmel SAML21 model [75]. It is a 32 bit RISC processor belonging to the ARM Cortex-M-0+ ultra-low consumption processor family. This microcontroller has a consumption of 35 μA/MHz in active mode and lower than 0.5 μA in standby mode when working with 3.3 Volts. It can perform several tasks at the same time. In this case, it allows values to be obtained from sensors in parallel, as well as storing and processing them for sending. Choosing the microcontroller for the device requires checking efficiency rankings, such as ULPMark [76]. The one selected for this purpose is the only microcontroller with ARM Cortex-0+ architecture included in this ranking. A development board, Sammy-L21 [77], was firstly employed for a first prototyping in this microcontroller, as shown in Figure 4.

Another key feature of this microcontroller is that it can perform customized logic operations due to its Configurable Custom Logic (CCL) unit [78]. This unit is really useful as it performs operations that allow it to compare a temperature value against the previous one measured, i.e., compare the rise and evaluate if it is a fast rise or not. Depending on these data, it may send an alert. This utility is created by using programmable and logical doors. It does not depend on a synchronous code snippet—it asynchronously and continuously works, even if the microcontroller is in sleep mode, thereby increasing the energetic efficiency.

Once the sensor has the relevant data and these data have been processed by the microcontroller, they are sent through a radio transceiver. The selected model for this purpose is a LoRa1276-C1, a sensor created by niceRF [79]. It has the relevant CE certification and provides great sound isolation as it isolates the radio sections from digital logic. In addition, it is worth noting its low consumption when in sleep mode, around 0.2 μA, which is an insignificant amount.

The entire system receives power from a reduced dimensions battery located in the lower section of the system. The battery capacity is defined by the requirements of the case study. The analysis of the device’s energetic consumption and the sizing of the required capacity are explained on the following pages.

### 3.3. WSN Energy Comparison and Selection

Urban areas have a lot of communication networks where the sensor network proposed in this work could be implemented. These networks are based on different radio technologies as the ones previously seen. Table 2 presents a comparison of the available technologies along with their relevant features. Features such as range, consumption and availability are emphasized because they are essential factors for developing the device.

In Table 2, MCL represents the minimum coupling loss, which means the minimum power through which communication between two devices is possible.

The range of each technology as well as its availability on the area where the sensor network is intended to be deployed are especially important in semi-urban or rural areas. Some technologies such as Wi-Fi ah or cellular cannot be applied when requiring a greater range (Wi-Fi) or when there is no availability (it depends on the cellular operator).

Power consumed by radio modules both in sleep mode and active mode to transfer or receive data from the network is another key factor when selecting the technology to be employed for the sensor network. Increasing the device’s battery life is crucial.

Considering these two factors, there are three options: Sigfox, LoRa and NB-IoT. On one hand, NB-IoT is currently dismissed due to its current availability and the high cost of its modules. On the other hand, Sigfox technology is only available on a subscription basis, which means an extra cost for the developed solution. Consequently, the technology selected for our system was LoRa.

Once the technology was selected, we decided to use LoRaWAN, which uses LoRa as a physical layer. LoRaWAN networks have previously been employed in the literature in other domains, for example, in an experimental performance evaluation of LoRaWAN over a real environment in Bangkok by Vatcharatiansakul et al. [80] and in a long range wide area network-based smart pest monitoring system by Yu et al. [81], among others [82].

Together with this decision, another important step to be considered was the selection of the LoRaWAN network server (and all the software involved). There were several options to deploy the LoRaWAN architecture which can be summarized in three options: (1) a free and open source option like LoraServer.io [83] which provides the software to implement the required features to be LoRaWAN compliant, specifically, the LoRa gateway bridge (packet forwarder), the LoRaWAN network server and the LoRaWAN application server. The maintenance and the hosting of the network server and some other application services are the responsibility of the user. (2) A free, open source and collaborative option like The Thing Network (TTN) [84] provides a self-hosted option, but also provides a free and collaborative platform as a service. That is, the platform is free, but every gateway using it can receive data from any node belonging to a TTN application. (3) A final option is choosing a complete solution offered by companies like Actility [46], Loriot [47] or Senet [48], among others. In some cases, they provide turnkey solutions for the infrastructure, data storing and even data analytics.

Table 3 presents options for the implementation of the LoRaWAN network server (and all the software involved) along with some of their features.

For our system, we selected TTN [84] as LoRaWAN the server network because of its collaborative deployment and free hosting option. This allows the use of the deployed network by all of the TTN community and provides a service that integrates with third-party applications at the same time. 

Consequently, the deployment of a LoRaWAN network benefits the entire area where the system is implemented. It provides the region with an infrastructure to develop applications based on this kind of technology and therefore, improves the technological development of the area. 

The system proposed in this work is decoupled from the LoRaWAN network server and application server so that any change in this element does not affect the other parts of the system.

### 3.4. Optimization Route Engine

**Figure 6:**
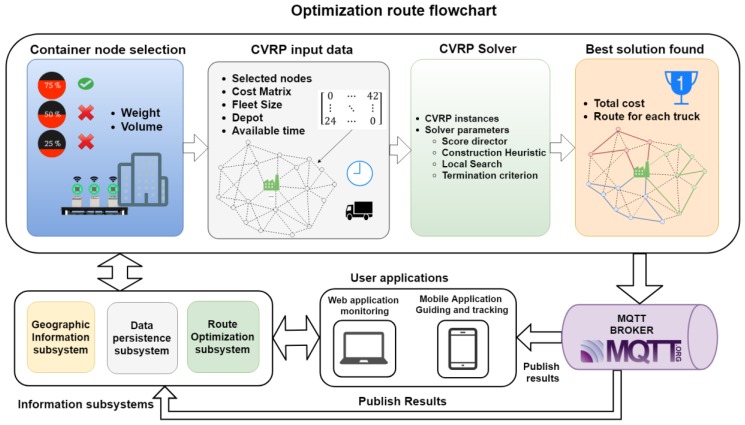
Optimization route flowchart.

The following pages focus on system section, related to the gathering, storing, processing and later use of sensors’ data. In reference to Figure 2 which shows information subsystems, Figure 6 indicates which of them are part of the route optimization for collecting waste.

The diagram located on the upper side presents the whole optimization process. It has four stages:

(1) Node selection: nodes visited by the waste collection fleet are selected. The criteria selection of nodes depends on the sensor variables at that moment—the weight and volume of each container. A threshold will be established to select the nodes that must be collected. This will depend on variables, such as current regulations of the town where the system is deployed [85] as well as waste intended to be collected. This is the reason why the criteria will change depending on the specific case study. When specifying the threshold, the following factors should be considered: filling frequency, data availability in the system and whether a criterion should be established or not, in accordance with the last waste report from the town. The criteria applied in this case study will be further explained later.
(2) CVRP data: once the nodes and the depot location have been obtained, the geographic information of every node is loaded from the geographical information subsystem to get the matrix of costs that will be employed for the CVRP resolution. The number of available vehicles must also be defined, as well as an ending criterion related to the time intended to be spent seeking the best solution. The system was implemented using the Graphhopper [86] framework and data from OpenStreetMap.
(3) CVRP solver: this is executed with previously indicated data in the route optimization subsystem. The heuristic construction of the solution and the algorithm of the local search are applied until the stop criteria is reached. A benchmark is employed to select the fastest optimization algorithm that can be used to obtain a feasible solution during the indicated time. Different construction heuristics are used for different local searches.
(4) Best solution found: once the best or most feasible solution is found (during the time specified under the stop criteria), it is published in the MQTT broker. The data persistence subsystem receives the information and stores the solution for that day’s collection plan. User applications (both mobile and web) will be notified through MQTT with the route that must be followed.

## 4. Case Study: Region of Salamanca

In order to validate the developed sensor and operating platform presented in this article, they are used in a case study in the region of Salamanca, Spain. The study is based on a public tender procedure from the year 2016 about the selective collection of waste (paper, packaging, etc.) [87]. According to the technical document, the regional administration of Salamanca requires the recollection of selective waste from 329 towns in the region and from their own installations. In addition, they include relevant information about number of containers in each town and their yearly productions of selective types of waste. In the same document, they specify a minimum fleet of eight vehicles for the collection.

**Figure 7:**
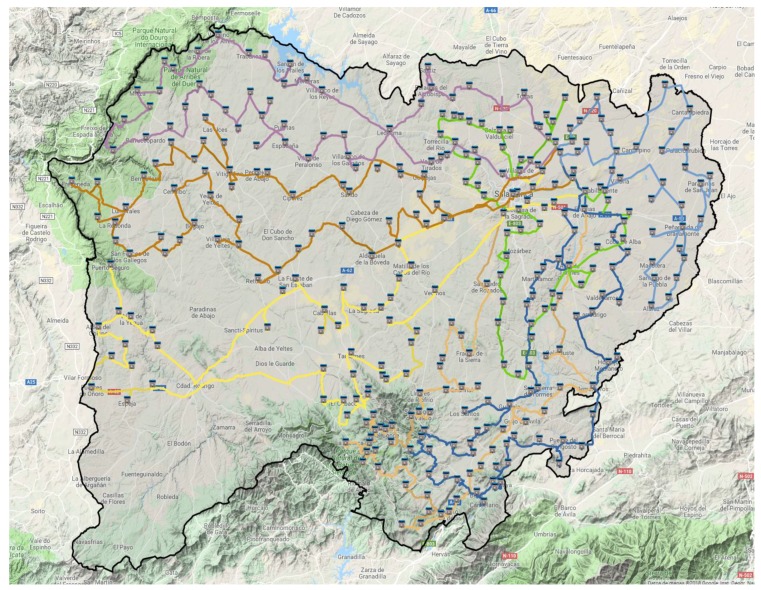
Towns of the case study and routes performed by the permanent vehicle fleet.

We used this information to formulate the CVRP problems. In the case study, we set the constraint that these vehicles collect waste every two weeks, and they always perform the permanent routes presented in Figure 7. This results in a cost of around 3050.725 km in journeys every two weeks. 

This case study was performed using synthetic data generated according to statistics of selective waste production in towns. This information was derived from the previous documents and the waste plan of the regional administration of Castilla y León (Junta de Castilla y León) [85]. Data related to the region in OpenStreetMaps was loaded in the geographical information subsystem.

The following section presents a discussion about the results related to the developed sensor, the WSN presented for this case study and the process of route optimization applied to this case study.

### 4.1. Measurements Results from Smart Sensors

A decisive factor of the proposed system is the nodes’ battery lives. One of the main purposes for this was to include the system without increasing the current maintenance requirements of containers, which must be revised between 1 and 4 times/year. This depends on the kind of waste and implies that the device battery must work for at least a year. The amount of battery life consumed by the node was evaluated by adding the power consumed by every built-in component and subsequently, the total consumption of the components together. 

Before creating the sensor, theoretical estimations of consumption were performed according to the datasheet of every component and the tools of their manufacture so that power consumption for each action could be calculated: sensor reading, information processing or sending through the network.

Different batteries in the market express the value using two quantities: power and electrical load, measured in Ah. One ampere/hour (Ah) is the electrical load unit that represents a current of one ampere passing through a conductive element for an hour. This allows the power of batteries to be described and consequently, the load that the device will consume for working can be calculated—expressed in mAh by Equation (9):

(9) $$Q=\underset{i}{\sum }i_{i}\cdot t_{i}=\;t_{MCUi}\cdot i_{MCUi}+t_{radio}\cdot i_{radio}+t_{MCUa}\cdot i_{MCUa}+\underset{j}{\sum }t_{sensorj}\cdot i_{sensorj.}$$

Q is the charge consumed in (mAh), $i_{i}$ is the power consumed by the microcontroller in idle mode and $t_{i}$ is the time. MCUa is the intensity of the microcontroller in active mode, and $i_{radio}$ is the intensity consumed by the radio module to perform the shipment. The summation presents consumptions of different j sensors during the capture of different data.

The values of consumption of each functional module in the node are summarized in Table 4 which is shown above.

The ultrasonic sensor generated a consumption of 15 mA when working, Due to its properties, the maximum operating time if the container is empty will consume 0.36 mAh.

The implemented weight system consisted of four load cells connected to a hub and an ADC amplifier which was accurate enough to capture the variation produced by an increase in weight. This amplifier provides the required voltage for the proper functioning of transducers. It only consumes 1.5 mA while performing a measurement for 40 ms. This means that the sensor uses 0.06 mAh per measurement. 

The temperature sensor employed for this case study (TMP36) was an analog temperature sensor. It consumes 4 mA and can perform measurements every 5 ms, so it uses 0.002 mAh per measurement.

The LoRa1276-C1 module—developed by niceRF—manages communications with the Gateway. This device uses 30 mA upon shipment and sends data in less than 10 ms, that is, the radio module consumes 0.3 mAh per shipment. 

These consumption numbers were added to the period of time that the microcontroller was active and fully working in order to obtain the total required power to read the sensors and perform the shipment.

The microcontroller uses 35 μA/MHz when working. As it works at a frequency of 48 MHz, it consumes 1.68 mA. In active mode, it performs two tasks: capturing data and managing shipments. For capturing data, the working time employed in the equation is the longest time spent obtaining measurements by sensors. In this case, the weight sensor takes the longest time to provide data—it provides a measurement in 40 ms. Consequently, the microcontroller takes 0.068 mAh in total to perform a measurement. When the MCU is in idle mode, it consumes 200 nA, so it uses a charge of 200 nAh (0.2 μAh) to be in idle mode for 1 h. Thus, we can confirm that we used 0.49 mA/measurement in total for capturing data.

However, we needed 10 ms—the time the communication module requires—for sending. The microcontroller uses 0.0168 mA during this period of time, so the shipment required 0.3168 mA in total.

Once the estimations were obtained, we proceeded to build the system and we obtained the battery consumptions. The tests consisted of simulations to define the real consumption rates of the node under different data capture and shipment conditions. This allowed us to specify the best rate to transfer data to the CVRP system and maintain a consumption level to keep the battery working for more than a year.

Table 5 describes some possible configurations of the nodes in order to estimate the consumption of the device. The first column describes the data shipment per day; these shipments are periodically performed in the same time intervals and provide the needed information for node selection.

The second column shows the number of measurements taken in a day. The third column displays the battery consumption estimated with the previously explained method. The fourth column shows the actual measured consumption of the device in the lab. 

### 4.2. Network Coverage Study

**Figure 8:**
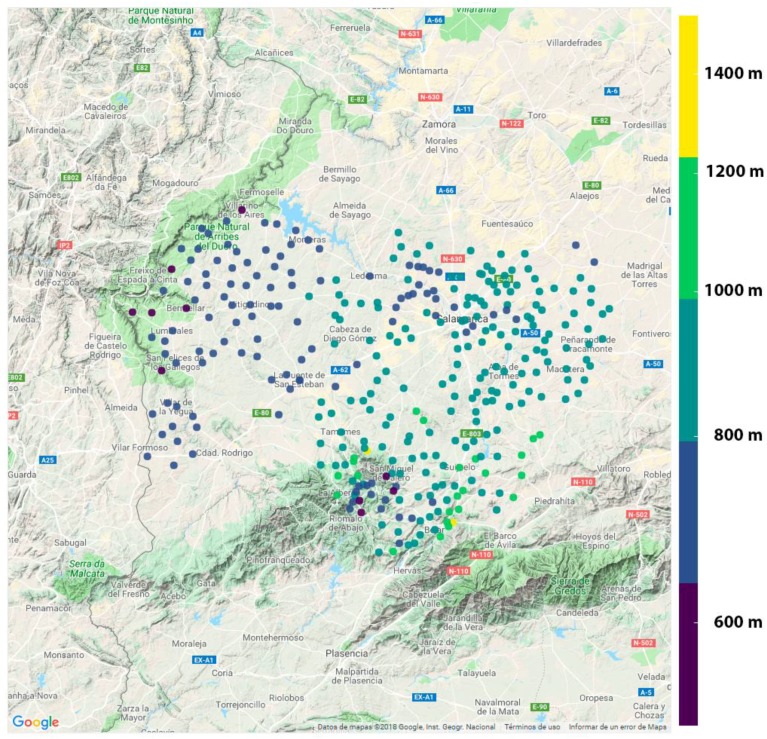
Towns in the case study and their corresponding altitudes in meters.

This section presents an initial study of the LoRaWAN coverage in the region of Salamanca. The terrain was extracted to identify the best locations for placing the antennas. The best options were towns with the highest altitudes in the area with easy-to-access internet to reduce costs. Figure 8 shows the height in meters of each town intended to receive coverage in the proposed case study.

**Figure 9:**
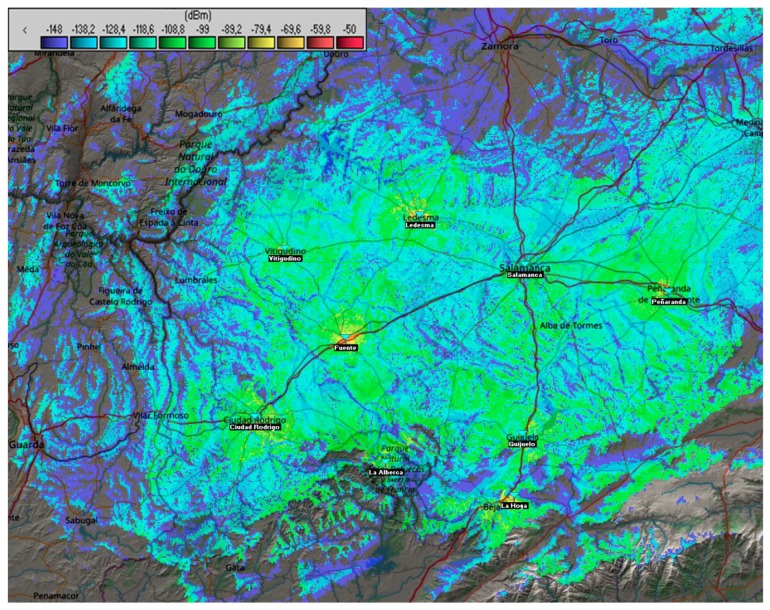
Coverage map of the study performed with the application, Radio Mobile.

Radio Mobile [88,89]—which is widely used to study radio frequency coverage—has been employed for studying the coverage. We have selected this tool over others like CloudRF [90] because it supports (just like CloudRF) the Longley–Rice propagation model (also known as Irregular Terrain Model ITM) and it is free to use. Based on previous data, we decided to deploy a network in the region consisting of nine Gateways, located, as shown in Figure 9, in Vitigudino, Ciudad Rodrigo and Fuente de San Esteban (West), La Alberca, La Hoya and Guijuelo (South), la Fuente de San Esteban (East) Ledesma (North) and Salamanca.

Figure 9 also shows the network coverage in dBm on the map. Using the terrain and the properties of LoRa technology, coverage can be provided to all the towns of the region as well as surrounding ones with no more than nine antennas in the area. 

This makes the deployment cost really low because several towns and their demarcations are covered by one Gateway, reducing the total cost of the deployment.

Variables employed in Radio Mobile to perform the coverage study are (1) for the base station, transmitter power of 0.5 W, receiver threshold of 0.0316 µV, line loss of 2.5, omnidirectional antenna, antenna gain of 13 dBi and a height of 20 m, and (2) for the nodes, transmitter power of 0.025 W, receiver threshold of 0.0316 µV, line loss of 0.1, dipole antenna, antenna gain of 2 dBi and a height of 1 m. These parameters were set according to the recommendations in ERC-REC-70-3E [91], European regulations regarding the radio frequency band of 868MHz which is a public bandwidth for European Low Power Networks (LPWAN).

The coverage map in Figure 9 is a view of merged Cartesian coverage where the transmission is specified by the node.

After the theoretical coverage study, several tests were performed in areas with lower coverage to test if the model employed in this case study was correctly adapted to real conditions. This resulted in the possibility of deploying a network in cases where rural areas use this technology, such as in smart farming domain works. Having this kind of infrastructure promotes the development of applications in this domain, even more if the deployed network includes LoRaWAN and The Things Networks and allows access to the network for the whole region.

### 4.3. Optimization Route Results

This section describes a simulation of the route optimization process using the data of waste production from the current waste plan of the regional administration of Castilla y León [85], the technical document of the public tender procedure of 2016 [87] and a report about selective collection of paper [92] which will be the waste collected in this case study. Waste collection scenarios were generated for 1 year with a total of 26 waste collection days. These scenarios were generated, taking into account the population of each town and the mean rate of waste production per inhabitant and day for each town.

In the simulation, we assumed that a visit to a town implies the collection of every container in that town; in this case study, the displacement produced by the truck in each town did not significantly penalize the total amount of kilometers of the route.

**Figure 10:**
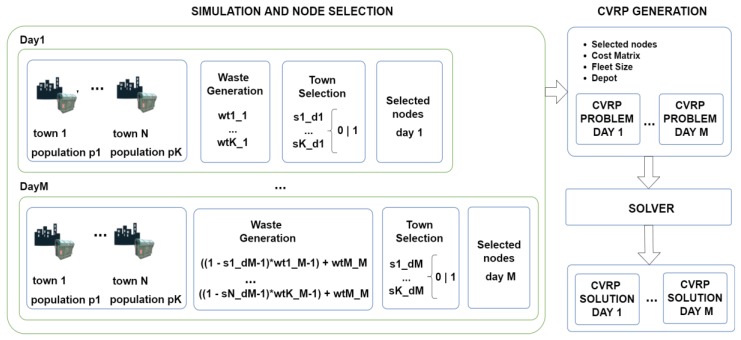
Simulation of waste, node selection, CVRP generation and solutions.

Figure 10 shows the simulation and selection of the towns on the left side. Firstly, the populations of all towns were obtained. Secondly, the waste generation was performed for each town to obtain a random value of waste that follows a normal distribution [93] considering the inhabitant waste generation ratio which has previously been mentioned:

(10) $$\left[W_{t11},\;\ldots ,\;W_{tK1}\right]=randomNormalValue\left(µ=townwaste_{t},\sigma =townwaste_{t}/10\right)$$

where $\left[W_{t11},\;\ldots ,\;W_{tK1}\right]$ is the waste generation of K towns for the first day of collection, and $townwaste_{t}=inhabitantWasteRatio*daysBetweenCollection*population_{t}$.

Thirdly, a selection node process was performed in order to include the towns considered for calculating the next waste collection routes. In this simulation, the variable employed was the weight of the waste produced by the whole town. However, in a real production environment the node selection would involve measurements of volume, and then, the weight of each container would be used for the definition of the CVRP problem. In this work, we only had data about the weight, so a estimation of volume using a paper density of 46.47 kg/m^3^ [92] was employed in the calculation. The selection criteria for each node was as follows:

(11) CurrentPopulationWaste+PopulationWasteUntilNextDay≥ 0.80∗PopulationWasteCapacity

where, in Equation (11), CurrentPopulationWaste is the total demand up to this day and PopulationWasteUntilNextDay is waste generation for the following days until the next collection day. If this is higher than the 80% of a population’s waste capacity, that population will be selected for collection. This percentage was extracted from the technical public tender document [87]. The PopulationWasteCapacity was calculated from the total population of the town and the number of containers available for that town. 

The previous process was performed for each collection day. The waste generation step considered the previous selection of nodes in order to add to the pending demand of every node which has not previously been visited.

As a result of this simulation process, the selected town nodes and their waste demands for the M collection days were obtained. The next step was generating the CVRP instances employing the selected nodes, the available vehicle fleet and the distance matrix of the selected town nodes.

Once the instances of CVRP were obtained, these were sent to the solver in order to solve it. The solve stage employed different heuristics and local search methods in order to find a feasible solution with the best score in a limited time of 24 h.

**Figure 11:**
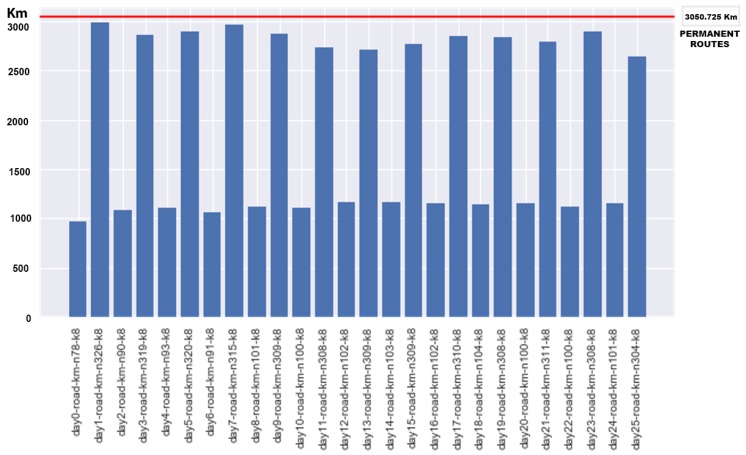
Results of each day of collection and the amount of km of fixed routes.

The results of the 26 collection days over a year are displayed in Figure 11. The distance in km are shown for each CVRP instance against the consumption of the permanent route approach.

**Figure 12:**
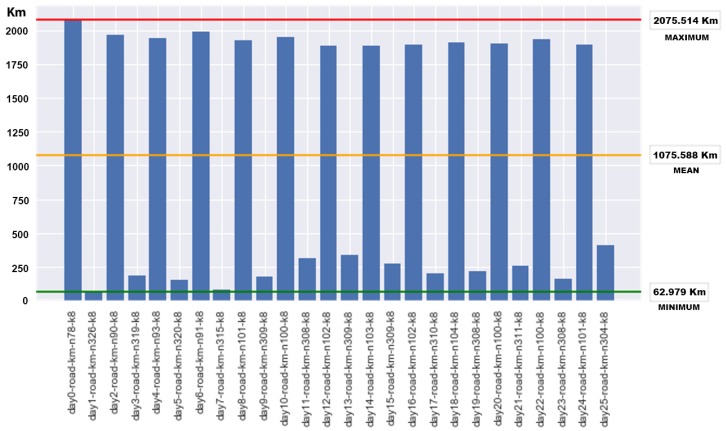
Savings results for each day of collection.

We can observe that the permanent routes have a cost of 3050.725 km (horizontal red line Figure 11) for each collection day whereas with the route optimization, as we expected, there are savings in the total cost of distance for all cases. Figure 12 shows the savings produced in each instance, with a maximum saving of 2075.514 km (upper red line in the chart) and a minimum saving of 62.979 km (bottom green line) and a mean saving of 1075.588 km (medium orange line) for each instance, which is a mean saving of 28%.

## 5. Conclusions and Future Works

The case study described in this work for waste collection in the region of Salamanca shows the improvement provided by the proposed system compared to a classic static collection approach. This system offers a better operation in terms of covered distance, fuel consumption, truck use and emissions, operating costs savings and collection of useful data for further waste generation analysis.

In regard to the developed sensor, it features a low consumption rate and sufficient autonomy to work throughout the presented case. The sensor correctly obtains data and sends them without penalizing the autonomy due to the employed radio technology.

Deploying this sensor allows the monitoring of implemented containers as well as the procurement of information about the waste production of inhabitants in each town. This helps the waste collection company detect whether more containers need to be deployed when the container filling is too fast at specific times of year, for example, when the population temporally grows during the summer. 

Regarding the coverage study, nine antennas in Salamanca region met the coverage requirements of the presented case study. This meant a reduced investment considering the covered extent of the land and the needed infrastructure in the region.

Concerning the route optimization results, the simulation offered mean savings in distance of 28% in the analyzed case. Due to these savings, the time and use of trucks are also decreased, and consequently, the workforce costs decreased. 

Finally, the simulation process of collecting paper and board waste along with obtained data showed that collecting waste every 2 weeks does not suit the waste production of every town in the community. The collection capacity is sometimes insufficient and in other cases the opposite. Selecting the indicated collection frequency is fundamental for saving time when performing this task. This is the reason why a system that dynamically generates routes with updated information provides a decision-making tool to perform collections with the proper frequency (when they are really needed).

In conclusion, as indicated by the case study, the proposed system includes a developed node which can operate efficiently for more than a year, obtaining useful data like weight, temperature and volume due to the low battery consumption. In addition, the coverage study showed that with a reduced investment, a deployment in the region with a small number of antennas covering the whole province of Salamanca is feasible. Together with these results, the optimization system results provide savings in cost, time and workforce against a static collection route approach.

The potential savings of totally deploying the waste collection system and the advantages of this kind of WSN could allow investment in the required infrastructure as well as enabling future IoT projects in the region, improving its technological development.

Future strands of work include the use of the sensor with the current waste collection company and the use of more data related to the waste collection which is provided by this company.

## Figures and Tables

**Table 1:** Comparison of volumetric sensors.

Sensor Type	Range (cm)	Accuracy (mm)	Angle of Operation (°)
Capacitive	0–3	3	5
Ultrasonic	20–400	8	20
Infrared	10–220	5	0
Radar	30–4000	15	0

**Table 2:** Wireless Sensor Network comparison.

Name	Minimum Coupling Loss (MCL) (dB)	Range (km)	Standby Consumption	Tx Consumption	Modulation	Availability
Random Phase Multiple Access (RPMA)	160	100	0.5 μA	85 mA	RPMA + DSSS	Spec. zones
Weightless P	128	2	0.7 μA	<70 mA	GMSK + QPSK	Worldwide
ZigBee	102	0, 20	3 μA	30 mA	BPSK	Worldwide
LoRa	157	5–15	0.5 μA	<90 mA	LoRa	Worldwide
Sigfox	149	3–10	0.5 μA	<70 mA	BPSK	Worldwide
Cellulars	118	2–5	10 mA	800 mA	8PSK	Worldwide
WiFi ah	90	<1	-	<100 mA	QPSK/256QAM	N/A
NB-IoT	118	2–5	5 μA	<100 mA	QPSK	Spec. zones

**Table 3:** Several LoRaWAN network server software options.

Name	Hosting	Open Source	Price Plan
LoraServer.io	Self-hosted	yes	free
The Thing Network (TTN)	Self-hosted/3rd party	yes	free/paid
Actility	3rd party	no	free (limited)/paid
Loriot	3rd party	no	free (limited)/paid
Senet	3rd party	no	free (limited)/paid

**Table 4:** Developed sensor consumption.

Sensor	Consumption (mAh)
Ultrasonic sensor	0.36
Load cells	0.06
Temperature sensor	0.002
Radio module	0.3
MCUi	0.0002
MCUa	0.068

**Table 5:** Measurements, shipments and consumption rates

Shipments/day	Measurements/day	Estimated Consumption (mAh per day)	Measured Consumption (mAh per day)
12	12	6.18	6.84
12	24	7.5	7.8
24	24	25.488	26.88
24	48	28.128	32.16
48	48	104.3232	107.04
